# Determining the Bulk Parameters of Plasma Electrons from Pitch-Angle Distribution Measurements

**DOI:** 10.3390/e22010103

**Published:** 2020-01-16

**Authors:** Georgios Nicolaou, Robert Wicks, George Livadiotis, Daniel Verscharen, Christopher Owen, Dhiren Kataria

**Affiliations:** 1 Department of Space and Climate Physics, Mullard Space Science Laboratory, University College London, Dorking, Surrey RH5 6NT, UK; r.wicks@ucl.ac.uk (R.W.); d.verscharen@ucl.ac.uk (D.V.); c.owen@ucl.ac.uk (C.O.); d.kataria@ucl.ac.uk (D.K.); 2 Southwest Research Institute, San Antonio, TX 78238, USA; george.livadiotis@swri.org; 3 Space Science Center, University of New Hampshire, Durham, NH 03824, USA

**Keywords:** space plasma, kappa distributions, pitch-angle distributions, plasma instruments

## Abstract

Electrostatic analysers measure the flux of plasma particles in velocity space and determine their velocity distribution function. There are occasions when science objectives require high time-resolution measurements, and the instrument operates in short measurement cycles, sampling only a portion of the velocity distribution function. One such high-resolution measurement strategy consists of sampling the two-dimensional pitch-angle distributions of the plasma particles, which describes the velocities of the particles with respect to the local magnetic field direction. Here, we investigate the accuracy of plasma bulk parameters from such high-resolution measurements. We simulate electron observations from the Solar Wind Analyser’s (SWA) Electron Analyser System (EAS) on board Solar Orbiter. We show that fitting analysis of the synthetic datasets determines the plasma temperature and kappa index of the distribution within 10% of their actual values, even at large heliocentric distances where the expected solar wind flux is very low. Interestingly, we show that although measurement points with zero counts are not statistically significant, they provide information about the particle distribution function which becomes important when the particle flux is low. We also examine the convergence of the fitting algorithm for expected plasma conditions and discuss the sources of statistical and systematic uncertainties.

## 1. Introduction

The environment of interplanetary space is filled with a very low-density plasma, primarily consisting of electrons and protons with a small component of heavier ions. Typically, investigations of this plasma measure the flux of particles in energy and angle to determine the velocity distribution function (VDF) of different particle species. The VDF indicates how energy is distributed between particles of the same type, and can be analysed accordingly to provide bulk properties of the plasma such as density, velocity, and temperature. The accuracy of the derived plasma bulk parameters depends on the accuracy of the measurements and on the techniques we use to analyse the data.

The velocities of space plasma particles often follow kappa distribution functions (see, e.g., in [1,2,3,4,5,6] and references therein). The kappa index labels and governs these distributions and it is another fundamental parameter that describes the thermodynamic state of the plasma. Theoretical works derived the kappa distribution from the foundations of Tsalis non-extensive statistical mechanics (see, e.g., in [7,8,9]). In addition, a large number of studies have determined kappa distribution functions in space plasma environments, such as the solar wind (see, e.g., in [10,11,12,13,14,15]), planetary magnetospheres (see, e.g., in [16,17,18]), cometary electrons (see, e.g., in [19]), and the outer heliosphere and inner heliosheath (see, e.g., in [20,21,22,23,24,25,26]).

The high energy “tails” that characterize kappa distributions are associated with relatively low particle fluxes, and are thus not always clearly resolved in plasma measurements. In these cases, the analysis becomes challenging, because a smaller number of particles are measured leading to a higher statistical uncertainty. Nevertheless, the accurate description of the plasma requires the determination of the kappa index [2,3,27]. Therefore, it is essential to obtain high-quality measurements that resolve the high-energy tails of the plasma distribution functions, and to use the appropriate analysis methods to determine the plasma bulk parameters from the observations.

Typical electrostatic analysers measure the flux of plasma particles over a finite range of energy and flow direction, at a given time. An almost complete scan through energies and directions is achieved by changing the voltages of the instrument components in a consecutive order. Space plasma is permeated by magnetic field which provides an important direction for anisotropy in the plasma. Charged particles gyrate around the magnetic field and particles can move relative to the magnetic field due to drift motions. The magnetic field is also an important direction for the propagation of plasma waves, wave-particle interactions, damping, heating and the generation of waves via instabilities from free-energy in the particle VDFs. Thus, in some operation modes, the measurement cycle of a plasma instrument is modified, and the instrument samples the particle energies in a limited range of directions only, providing the measurements to construct the two-dimensional (2D) pitch-angle distribution function of the plasma. Although the reduction by one dimension allows faster scans which provide high time-resolution measurements, it reduces the statistical significance of the data because the distribution is resolved in fewer points in velocity space.

For example, the Plasma Electron and Current Experiment (PEACE) instrument [28] on board Cluster is designed to measure the plasma electrons within the Earth’s magnetosphere. PEACE consists of two top-hat electrostatic analysers, with look directions onto a plane perpendicular to the spin axis of the spacecraft. During the nominal operation mode, PEACE constructs the three dimensional (3D) VDF of the plasma electrons over the energy range from 0.59 eV to 26.4 keV. The 3D VDF can be constructed over half a spin period of the spacecraft, which corresponds to 2 seconds. In cases when the magnetic field direction lies within the instrument’s azimuthal field of view, we can construct the pitch angle distribution of the plasma electrons over a time period of just 62.5 milliseconds—the time it takes for the instrument to scan in energy.

As another example, the Solar Wind Analyser’s Electron Analyser System (SWA-EAS [29]) on board Solar Orbiter, will measure the solar wind electrons in the energy range from 1 eV to 5 keV, within heliocentric distances from ~0.3 to 1 au. In its nominal operation mode, SWA-EAS completes an energy-direction scan constructing the entire 3D VDFs of the plasma electrons in ~1 s. In burst-mode, the instrument will measure the 2D pitch-angle distribution of the electrons over a period of 0.125 s, using a single deflection state. The accuracy of the derived plasma bulk parameters is a function of the electron flux, which depends on the solar wind density, and speed. Since Solar Orbiter will observe plasma within a wide range of heliocentric distances and heliographic latitudes, we expect a wide range of plasma fluxes to be sampled.

In this study, we investigate the accuracy of our derivation of the plasma bulk parameters, such as the plasma density, temperature tensor and kappa index, from the analysis of the expected 2D pitch-angle measurements by SWA-EAS on board Solar Orbiter. We model the expected measurements of solar wind plasma electrons, considering the instrument’s ideal response, based on the initial instrument calibration. We then fit the synthetic dataset with an analytical model in order to derive the bulk parameters of the electrons. We compare the derived parameters with those from our input. Through this comparison, we quantify the accuracy of the derived parameters as a function of the recorded counts. In Section 2, we describe in detail our instrument model and how we model and analyse the expected observations. In Section 3, we present our results, which we discuss in detail in Section 4. Section 5 summarises our conclusions.

## 2. Methods 

### 2.1. Instrument Model

**Figure 1:**
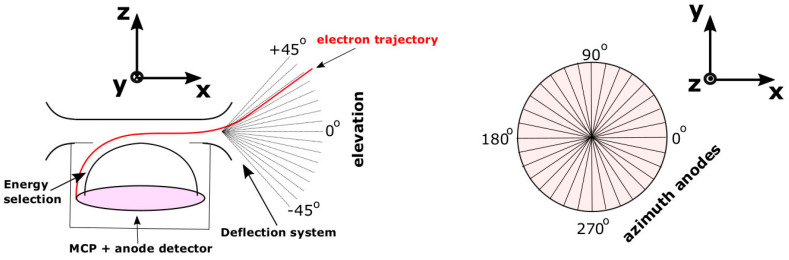
Schematic of a Solar Wind Analyser’s Electron Analyser System (SWA-EAS) top-hat analyser head and its angular field of view. (**Left**) The elevation angle is defined as the complement of the angle between the particle velocity vector and the *z*-axis, perpendicular to the top-hat plane. The elevation angle of the electrons is resolved in 16 electrostatic uniform steps. (**Right**) The azimuth angle is the angle within the projection of the velocity vector on the top-hat plane and the *x*-axis. Both SWA-EAS analyser heads resolve the azimuth direction on MCP detectors using 32 sectors.

SWA-EAS consists of a pair of top-hat electrostatic analyser heads, orthogonally mounted on the spacecraft boom. Each analyser head includes an aperture deflector system and a position-sensitive multichannel plate (MCP) detector. The instrument measures the plasma electron fluxes as a function of kinetic energy and angle of incidence from which the electron VDF is calculated. SWA-EAS scans the energy of the electrons by electrostatic selection in 64 steps, spread exponentially over the range between 1 eV to 5 keV. Each specific kinetic energy step *E* has a width of Δ*Ε*/*Ε* ~ 12.5%. In the nominal operation mode, the aperture deflector system of each analyser head uses voltages applied to plates at the aperture entrance to deflect incoming electrons and scans through the elevation direction of the plasma particles, defined as the complement of the angle within the particle velocity vector and the *z*-axis perpendicular to the top-hat aperture plane (left panel of Figure 1). Each of the two SWA-EAS analyser heads measure specific elevation directions *Θ* within the range from −45° to +45° in 16 uniformly spaced electrostatic steps. We model an ideal response in which each *Θ* is measured with a resolution of Δ*Θ* = 6°. The azimuth direction is the angle within the projection of the velocity vector on the top-hat plane (*x*-*y* plane) and the *x*-axis (right panel of Figure 1). Each SWA-EAS analyser head resolves 32 discrete azimuth directions *Φ* covering the full range from 0° to 360° by 32 azimuth sectors mounted on the MCP. Each azimuth direction *Φ* has a resolution of Δ*Φ* = 11.25°. Combining two of the analyser units offset by 90° allows the full sky to be measured. Thus, one analyser can always see the direction towards the magnetic field vector $\vec{B}$. In burst-mode operations, the analyser head that can see $\vec{B}$ performs two consecutive energy scans; one with the aperture deflectors set to *Θ* = *θ_B_*, followed by a scan with the deflectors set to *Θ* = −*θ_B_*, where *θ_B_* is the elevation angle of the magnetic field vector, which has been recorded by the magnetometer on board Solar Orbiter [30] just before the EAS scan. Thus, the flux of electrons will be measured in two cones, one with an edge aligned along and one anti-parallel with $\vec{B}$. From these measurements a pitch-angle distribution can be recovered.

### 2.2. Synthetic Dataset

We use the well-established forward-modelling technique (see, e.g., in [31,32,33,34,35,36,37,38,39]) to simulate the expected SWA-EAS observations in the solar wind. We focus on the characterization of supra-thermal electrons, which we consider more challenging due to the high energy tails of their VDFs. We model solar wind electrons with their velocities following the bi-kappa distribution function (see, e.g., in [3,4,5,6])

(1) $$f(\vec{u})=\frac{n}{\sqrt{T_{∥}}T_{⊥}}{\left[\frac{m_{e}}{2\pi \left(\kappa -3/2\right)k_{B}}\right]}^{3/2}\frac{\Gamma (\kappa +1)}{\Gamma (\kappa -1/2)}{\left[1+\frac{m_{e}{\left(u_{∥}-u_{0,∥}\right)}^{2}}{2k_{B}\left(\kappa -3/2\right)T_{∥}}+\frac{m_{e}{\left({\vec{u}}_{⊥}-{\vec{u}}_{0,⊥}\right)}^{2}}{2k_{B}(\kappa -3/2)T_{⊥}}\right]}^{-\kappa -1},$$

where *n* is the electron number density, $u_{∥}$, ${\vec{u}}_{⊥}$ are the particle velocity parallel and perpendicular to the magnetic field respectively and $u_{0,∥}$ and ${\vec{u}}_{0,⊥}$ are the corresponding bulk velocities. $T_{∥}$, and $T_{⊥}$ are the parallel and the perpendicular temperature, respectively, and *κ* is the kappa index. Finally, Γ is the gamma function, k_B_ is the Boltzmann constant and m_e_ is the electron mass.

We consider a simple set-up with $\vec{B}$ in the top-hat plane of an individual SWA-EAS analyser head (magnetic field elevation angle *θ*_B_ = 0°) and the analyser head performing a single scan through kinetic energy *E* and azimuth *Φ*, for elevation *Θ* = *θ*_B_ = 0°, with acquisition time Δ*τ*. The instrument records an expected number of counts,

(2) $$C_{\exp}(E,\Theta =\theta_{B}=0,\Phi )=\frac{2}{m_{e}{}^{2}}Gf(E,\Theta =\theta_{B},\Phi )E^{2}\Delta \tau ,$$

where

(3) $$G=A_{\mathrm{eff}}\frac{\Delta E}{E}\Delta \Theta \Delta \Phi ,$$

is the geometric factor of the instrument and *A*_eff_ is the effective area, which is a function of the instrument’s aperture and the detector’s efficiency. For this study, we assume a constant *A*_eff_ which results in a constant *G*, although in reality, both could be functions of energy, elevation and azimuth. For further simplification, we only consider cases in which the bulk velocity vector points towards the *x*-axis, so that the elevation and the azimuth angle of the bulk velocity vector *θ*_u,0_ = φ_u,0_ = 0°. We also set the azimuth angle of the magnetic field to *φ*_B_ = 45°. We note that in this specific set-up, the bulk speed components have the same magnitude $\left|{\vec{u}}_{0,∥}\right|=\left|{\vec{u}}_{0,⊥}\right|$, and the azimuth sectors of the instrument measure the pitch-angles of the particles. We assume that the registered counts *C* follow the Poisson distribution:

(4) $$P(C)=e^{-C_{\exp}}\frac{C_{\exp}{}^{C}}{C!}.$$

**Figure 2:**
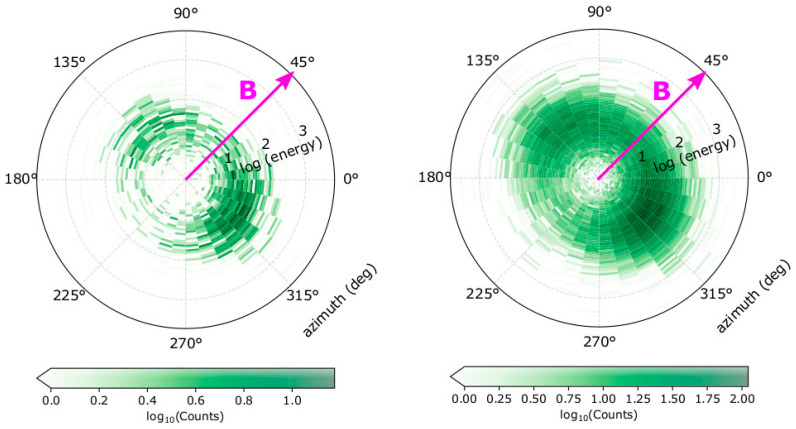
Modelled counts as a function energy and azimuth direction on the analyser’s head frame for (**left**) plasma density *n* = 5 cm^−3^ and (**right**) *n* = 50 cm^−3^. For both examples, the magnetic field vector (magenta) is in the top-hat plane (*Θ* = *θ*_B_ = 0°) in azimuth direction *Φ* = 45°. The bulk flow of the electrons *u*_0_ = 500 kms^−1^ along the *x*-axis (*Θ* = *Φ* = 0°). The parallel temperature $T_{∥}$ = 10 eV, the perpendicular temperature $T_{⊥}$ = 20 eV, and the kappa index *κ* = 3.

In Figure 2, we show two examples of the synthetic datasets, based on electron plasma densities of *n* = 5 cm^−3^ (left panel), and *n* = 50 cm^−3^ (right panel). For both examples, the bulk velocity of magnitude *u*_0_ = 500 kms^−1^ points along the *x*-axis, and we set *κ* = 3, $T_{∥}$ = 10 eV, and $T_{⊥}$ = 20 eV. 

In our approach we neglect effects related to the spacecraft potential. In reality, the spacecraft floats at a potential caused by interaction with the space environment and depending on the composition of materials that cover the surface of the spacecraft. This potential can be of order 10 V or more. Furthermore, UV photons striking the spacecraft produce photoelectrons that form a cloud around the spacecraft. Thus, the incoming solar wind electron population can be accelerated by a few eV and obscured by a high-density population of electrons at all energies below the spacecraft potential. Furthermore, it is possible for the VDF of the electrons to have an irregular shape in 3D. We ignore these effects here as we are interested in the high-energy tail of the electron distribution which should not be affected by these phenomena, and we assume gyrotropy, i.e., that the VDF is symmetrical in rotation around the magnetic field direction.

**Figure 3:**
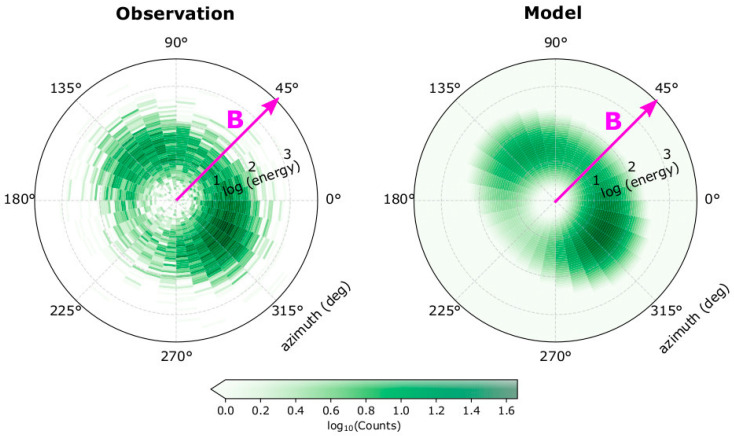
(**Left**) Modelled counts as a function of energy and azimuth direction (instrument frame), using *n* = 20 cm^−3^, *u*_0_ = 500 kms^−1^ towards the *x*-axis (*Θ* = *Φ* = 0°), *κ* = 3, $T_{∥}$ = 10 eV, $T_{⊥}$ = 20 eV, and a magnetic-field direction (magenta) in the top-hat plane (*Θ* = 0° and *Φ* = 45°). (**Right**) Result of our fit to the modelled observations. The model finds the optimal combination of *n*, *κ*, $T_{∥}$, and $T_{⊥}$ that minimizes the *χ*^2^ value (see text for more).

We derive the plasma properties by fitting our analytical model of the expected counts in Equation (2) to the observations. During the actual operations of SWA-EAS, $\vec{B}$ will be determined from Solar Orbiter’s magnetometer instrument [30]. Therefore, in our fitting analysis, the parallel and perpendicular directions are known parameters. Moreover, we reduce the unknown parameters of the fitted model by obtaining the plasma bulk velocity vector from proton measurements by the Proton Alpha Sensor (SWA-PAS [29]) on board the same spacecraft. This approach assumes that protons and electrons have the same bulk velocity, which is generally true for plasma with zero net charge and no significant drifts among the ion species. We note, however, that a more sophisticated approach could leave the electron bulk velocity as a free parameter to be derived by the fitting analysis. We then use a chi-squared minimization algorithm which defines the optimal combination of the undetermined plasma parameters (*n*, *κ*, $T_{∥}$, and $T_{⊥}$) that minimize the chi-squared value

(5) $$\chi^{2}=\frac{1}{N-R}\sum_{i=1}^{N}{\left[\frac{C_{i}-C_{\exp,i}(n^{\prime },\kappa^{\prime },T_{∥}^{\prime },T_{⊥}^{\prime })}{\sigma_{i}}\right]}^{2},$$

where *N* is the total number of data-points we consider for fitting, *R* is the number of the model’s free parameters, and *σ_i_* is the standard deviation of each count *C_i_*. The model we fit to the observations is a function of *n*, *κ*, $T_{∥}$, and $T_{⊥}$, so *R* = 4. In plasma applications, we typically use $\sigma_{i}=\sqrt{C_{i}}$, as within a complete scan, we obtain only one measurement for each specific point in velocity space, and we assume that it represents the average value and the variance of the expected number of particles. For each measurement sample, we perform two fittings; one which excludes all points with *C_i_* = 0, and one which includes all points with *C_i_* = 0 in the analysis. We note that, in the second type of fitting, we assign an uncertainty of *σ_i_* = 1 to each *C_i_* = 0 measurement (see, e.g., in [40]). In Figure 3, we show an example of our synthetic dataset and the corresponding model fitted to it. (The software used in this study is available for download at www.github.com/gnicolaou85/Elfit)

## 3. Results

**Figure 4:**
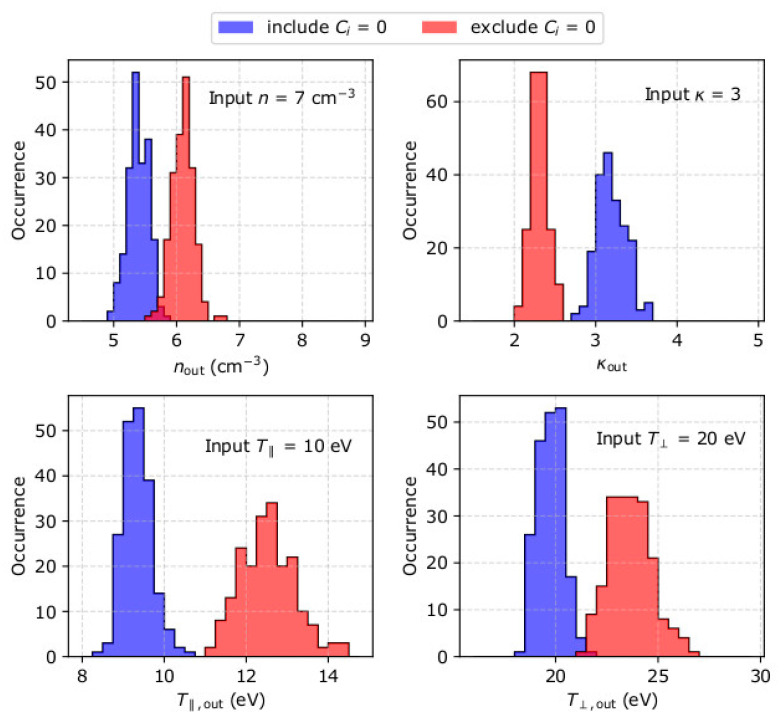
Histograms of (**top left**) density *n*_out_, (**top right**) kappa index *κ*_out_, (**bottom left**) parallel temperature $T_{∥,\mathrm{out}}$ and (**bottom right**) perpendicular temperature $T_{⊥,\mathrm{out}}$, as determined from the analysis of 200 measurement samples of plasma with *n* = 7 cm^−3^, *u*_0_ = 500 kms^−1^ pointing along the *x*-axis, *κ* = 3, $T_{∥}$ = 10 eV and $T_{⊥}$ = 20 eV. The blue histograms correspond to values derived by a fit that includes points with *C_i_* = 0, while the red histograms represent values derived by a fit that excludes points with *C_i_* = 0.

For each set of input plasma conditions, we simulate 200 measurement samples which we fit to derive the electron density, temperature and kappa, as explained in Section 2. By investigating the histograms of the derived parameters, we verify that 200 samples are sufficient to derive statistically significant results, especially within the low density (low particle flux) range. The histograms of the derived parameters from the analysis of 200 samples with input parameters *n* = 7 cm^−3^, *u*_0_ = 500 kms^−1^ pointing along the x-axis, *κ* = 3, $T_{∥}$ = 10 eV, and $T_{⊥}$ = 20 eV, are shown in Figure 4. Table 1 shows their average values and standard deviations. For these input plasma conditions, the average derived plasma density is by ~23% lower than the input plasma density if the fitting includes *C_i_* = 0, and by ~13% lower than the input density when *C_i_* = 0 are not included in the fit. The standard deviation of the derived densities is $\sigma_{n}$ ~0.2 cm^−3^ for both fits. On average, the fitting analysis that includes *C_i_* = 0 overestimates the kappa index by ~7%, while the fitting that excludes *C_i_* = 0 underestimates kappa by ~23 %. The standard deviations of the derived kappa indices are ~ 0.2 and 0.1 respectively. The average derived $T_{∥}$ is by ~6% lower than the actual value when *C_i_* = 0 are included in the fit, and by ~25% larger than the actual value when *C_i_* = 0 are not included in the fit. The standard deviation of $T_{∥}$ is 0.3 eV when *C_i_* = 0 are fitted and 0.7 eV when *C_i_* = 0 are not fitted. The fit that includes *C_i_* = 0 derives accurately $T_{⊥}$ within the standard deviation $\sigma T_{⊥}$ = 0.6 eV. The fit that excludes *C_i_* = 0 from the fit overestimates $T_{⊥}$ by ~19%, with standard deviation $\sigma T_{⊥}$ = 1 eV.

**Figure 5:**
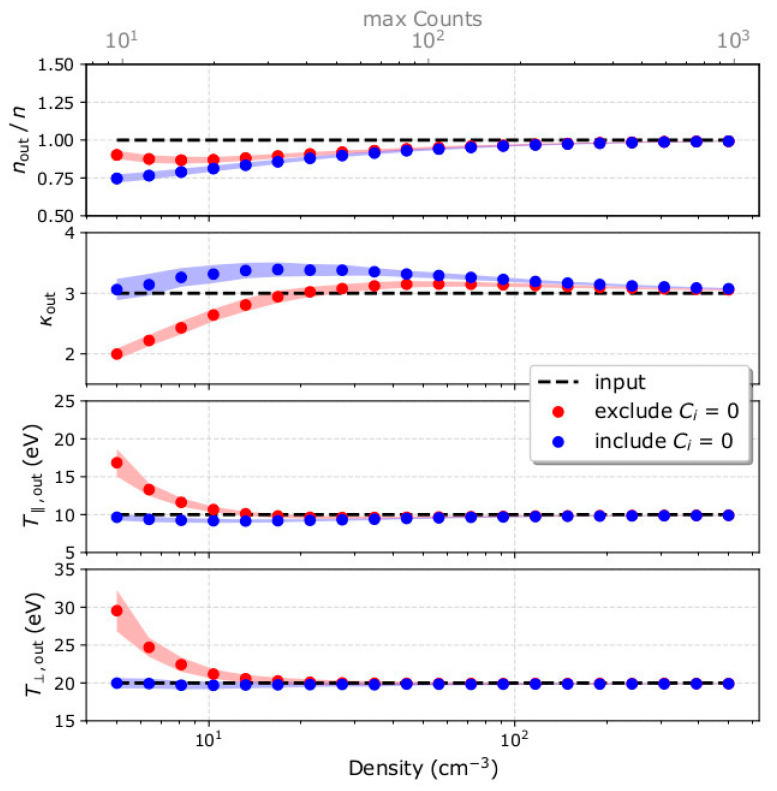
(**From top to bottom**) The derived electron density over input density, kappa index, parallel and perpendicular temperature as functions of the input plasma density. The red points represent the mean values (over 200 samples) of the parameters derived by fitting only the measurements with *C_i_* ≥ 1. The blue points represent the mean values of the parameters derived by fitting to all measurements including those with *C_i_* = 0. The shadowed regions represent the standard deviations of the derived parameters.

The solar orbiter expects densities between ~5 cm^−3^ and ~500 cm^−3^. In Figure 5, we show the derived parameters as functions of the plasma density over the expected range. The red points show the average (over 200 samples) values of the derived parameters as determined by our fits excluding points with *C_i_* = 0, and the blue points show our results for the fit analysis including points with *C_i_* = 0. The shadowed regions represent the standard deviations of the corresponding values. The horizontal axis on the top shows the maximum number of the expected counts (peak value at an individual *E*, *Φ*) for the specific input parameters. On the lower side of the density (and counts) range, the plasma density is underestimated in both fitting strategies; however, it is more accurately determined by both fitting strategies when *n* > 50 cm^−3^ (*C*_max_ > 100). 

The kappa index is significantly misestimated (up to 35%) over the low input density range, if the analysis excludes points with *C_i_* = 0. For instance, when *n* = 5 cm^−3^, the derived index *κ*_out_ ~ 2. Interestingly, for the same plasma, *κ*_out_ is accurately determined when *C_i_* = 0 measurements are included in our fit. However, as the density increases to *n* > 20 cm^−3^, the kappa index is more accurately determined when derived by fits excluding points with *C_i_* = 0.

For the input parameters we examine, the fitting analysis that excludes points with *C_i_* = 0 significantly overestimates the plasma temperature in the low-density range (*n* < 10 cm^−3^). For instance, when *n* = 5 cm^−3^ (*C*_max_ ~ 10), the derived $T_{∥}$ ~ 17 eV, which is 1.7 times greater than its actual input value, and the derived $T_{⊥}$ ~ 30 eV, which is 1.5 times greater than its actual input value. For the same plasma conditions, when the analysis includes points with *C_i_* = 0, the derived temperatures do not deviate from their actual values by more than 4 %. Nevertheless, the two fitting analyses determine similar temperatures for *n* > 10 cm^−3^.

**Figure 6:**
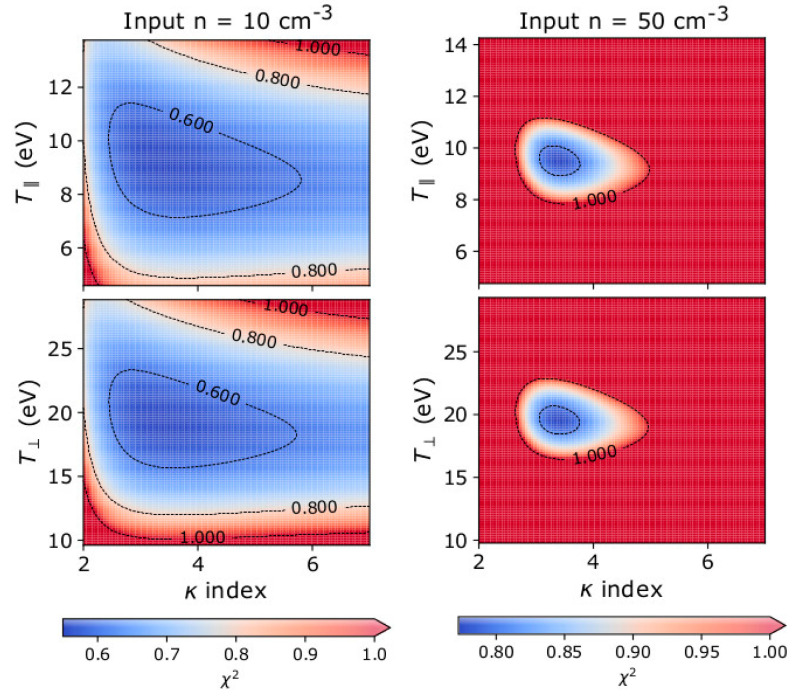
2D histograms of the *χ*^2^ value as a function of (**top**) the modelled *κ* and $T_{∥}$ and (**bottom**) the modelled *κ* and $T_{⊥}$, as derived for plasma with two different input densities; (**left**) *n* = 10 cm^−3^, and (**right**) *n* = 50 cm^−3^. In both examples, we use *u*_0_ = 500 kms^−1^ pointing along the x-axis, *κ* = 3, $T_{∥}$ = 10 eV, and $T_{⊥}$ = 20 eV as input parameters.

Finally, we examine the fit convergence as a function of the plasma parameters; the minimum *χ*^2^ defines the best fit parameters. Previous studies [27,33] show that the accurate estimation of the plasma temperature depends on the accurate determination of the kappa index. In Figure 6, we show 2D plots of the *χ*^2^ value as a function of the model parameters *κ*, $T_{∥}$, and $T_{⊥}$. For each panel, we show *χ*^2^ as a function of two parameters at a time, and we keep the remaining model parameters to their values as determined by the best fit. We perform our calculations for input plasmas with two different densities: *n* = 10 cm^−3^, and *n* = 50 cm^−3^. In both examples, we use *κ* = 3, *u*_0_ = 500 kms^−1^ pointing along the x-axis, $T_{∥}$ = 10 eV, and $T_{⊥}$ = 20 eV as the input parameters. Red areas on the plots in Figure 6 indicate *χ*^2^ > 1. For a fixed acquisition time Δ*τ* and fixed geometric factor *G*, higher densities result in higher counts and a smaller area of low *χ*^2^, which indicates that the derived parameters possess a smaller uncertainty. The non-axisymmetric shape of the area of low *χ*^2^, indicates an interdependency of the derived kappa index and the temperatures. More specifically, the area of low *χ*^2^ shifts towards higher temperatures and gets broader along the vertical axis for smaller *κ*.

## 4. Discussion

We examine the accuracy of plasma electron bulk parameters as derived from the analysis of the expected observations by SWA-EAS operating in burst-mode. Generally, the accuracy of the derived parameters is a function of the flux of plasma particles (number of recorded counts). Our analysis shows that the fit analysis of samples with a significant amount of counts (*C*_max_ > 30), derives accurately the plasma parameters when measurement points with zero counts (*C_i_* = 0) are excluded from the analysis. We show that when analysing observations with a low amount of counts (*C*_max_ < 30), the accuracy of some parameters is improved if measurement points with zero counts are included in the fitting analysis. 

The uncertainty of each measurement *C_i_* is approximated with $\sigma_{i}=\sqrt{C_{i}}$, assuming that the recorded signal follows the Poisson distribution. This results to a relative uncertainty $\frac{\sigma_{i}}{C_{i}}=\frac{1}{{\sqrt{C}}_{i}}$ which becomes considerably large for small *C_i_* and propagates significant statistical and systematic errors in the derivation of the plasma bulk properties. Although Poisson statistics does not really apply to *C_i_* = 0, these bins indicate points of velocity space with low fluxes which do not reach the detection threshold. This information is still useful, especially when the overall signal is weak.

**Figure 7:**
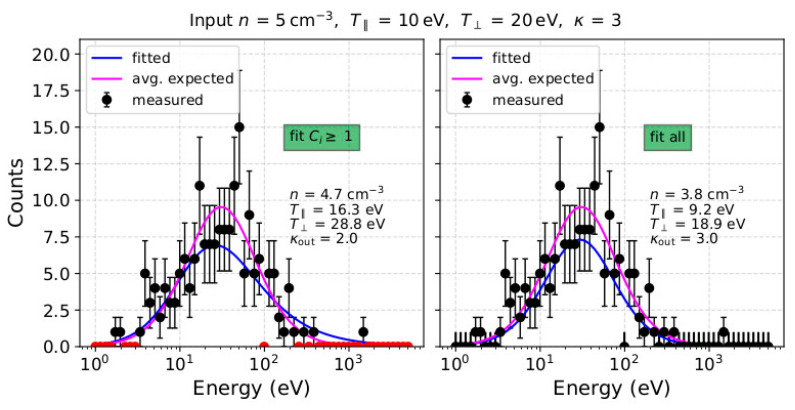
Number of counts as a function of energy for the pitch-angle with the maximum flux assuming a plasma with *n* = 5 cm^−3^, *κ* = 3, $T_{∥}$ = 10 eV and $T_{⊥}$= 20 eV. The blue line is the fitted model to the observations by (**left**) excluding points with *C_i_* = 0 which are shown with red colour, and (**right**) including points with *C_i_* = 0. The magenta line is the expected counts *C*_exp_, given by Equation (2). The labels in each panel show the parameters as derived by the corresponding fit.

In Figure 7, we show the distribution of counts as a function of energy, recorded in the azimuth anode which contains the maximum number of counts *C*_max_, assuming a plasma with *n* = 5 cm^−3^, *κ* = 3, $T_{∥}$ = 10 eV and $T_{⊥}$ = 20 eV. The blue curves in both panels show the analytical model fitted to the data (fitted to the 2D distribution of counts) and the magenta curve indicates the expected number of counts. In the left panel, the fitting analysis excludes measurements with *C_i_* = 0 (red points), whereas in the right panel, the fitting analysis includes measurements with *C_i_* = 0 with standard deviation *σ_i_* = 1. The kappa index of the fitted model in the left panel is ~2, and the distribution’s high-energy tail is prominent up to 2 keV, based on the inclusion of the non-zero data-points beyond ~400 eV, neglecting all points with *C_i_* = 0 in between. On the other hand, the fit result in the right panel does not have a prominent tail extending beyond 400 eV, as it fits all *C_i_* = 0 points within that energy range. In this case, *κ*_out_ ~ 3 which is an accurate estimation of the actual kappa index of the modelled plasma.

**Figure 8:**
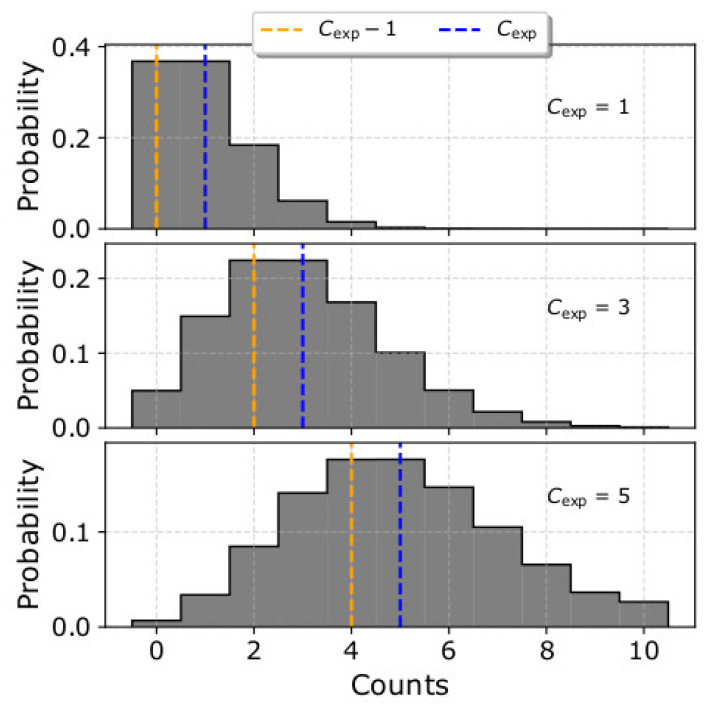
Poisson distribution with average value (**top**) *C*_exp_ = 1, (**middle**) *C*_exp_ = 3, and (**bottom**) *C*_exp_ = 5. The vertical lines indicate the two modes of the distribution, *C*_exp_ (blue) and *C*_exp_ − 1 (orange) respectively. For small average values, the Poisson distribution is asymmetric, and the probability to measure number of counts lower than the average value is significant. This can bias the results to lower densities.

**Figure 9:**
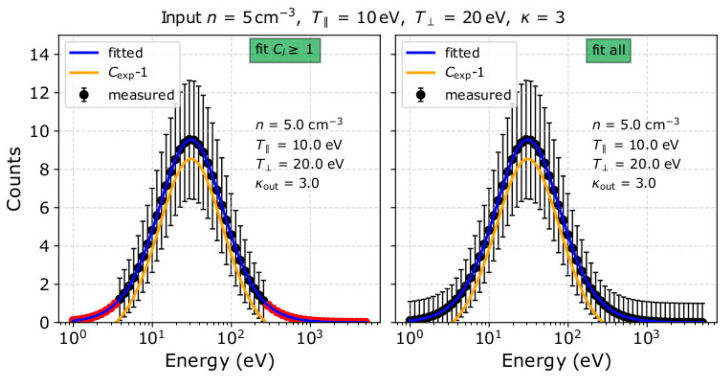
Number of the expected average counts *C*_exp_ as a function of energy in the pitch-angle bin with the maximum particle flux, considering the same plasma conditions as in the example shown in Figure 7. The blue curve is the model fitted to the observations by (**left**) excluding points with *C_i_* = 0 which are shown with red colour, and (**right**) including points with *C_i_* =0. The orange curve is the mode *C*_exp_ − 1. In each panel, we show the parameters as derived by the corresponding fit. In the absence of statistical fluctuations, both fitting strategies derive identical bulk parameters.

As we show in Figure 5 and Figure 7, both fitting strategies underestimate the plasma density when the input density is *n* < 50 cm^−3^. This is partially due to the asymmetry that characterizes the Poisson distributions of low counts. In Figure 8, we show the Poisson distributions for *C*_exp_ = 1, *C*_exp_ = 3 and *C*_exp_ = 5. Each distribution has two modes (most frequent values); the higher mode which is equal to the average value of the distribution *C*_exp_, and the lower mode = *C*_exp_ − 1. The relative difference between the two modes increases with decreasing *C*_exp_. Samples drawn from a Poisson distribution with a small *C*_exp_, as is the case at low densities expected to be measured by SWA-EAS at ~1 au, will more likely undersample the distribution than oversample, and so densities will be underestimated. In addition, within a measurement cycle, each point of velocity space is sampled only once. In our analysis, we consider that an individual *C_i_* obtained at a specific point of velocity space is representative of the average value *C*_exp_ with uncertainty $\sigma_{i}=\sqrt{C_{i}}$. This introduces an additional systematic error. We illustrate this error by considering an exemplar Poisson distribution with *C*_exp_ = 5. If we obtain one measurement, the probabilities for observing *C_i_* = 1 and *C_i_* = 9 are almost the same (see Figure 8). However, in our fitting analysis, the corresponding uncertainties for *C_i_* = 1 and *C_i_* = 9, are *σ_i_* = 1 and *σ_i_* = 3, respectively, and a *χ*^2^ minimization model fitted to these two points will shift towards *C_i_* = 1, as the specific point has a bigger weight $\sigma_{i}^{-2}=C_{i}^{-1}=1$. This specific systematic error purely depends on the statistical uncertainty of the measurements. We prove this by fitting the mean values of the Poisson distribution (or higher mode value) with both fitting strategies (Figure 9). For the specific example, we consider plasma with the same bulk properties as in the example shown in Figure 7 and we show that in the absence of statistical fluctuations, both fits derive identical results. The orange curve in both panels is the lower mode of the Poisson distribution *C*_exp_ − 1, which corresponds to a distribution with lower density. In reality, the errors associated with the statistical uncertainty of the measurements decrease with increasing number of counts and/or when analysing average counts over multiple samples of the same plasma. 

## 5. Conclusions

We investigate the accuracy of the electron plasma bulk parameters as derived by our analysis of SWA-EAS high-time resolution measurements. We assume electrons with velocity distribution function following the anisotropic bi-kappa distribution function and simulate the expected counts in the instrument’s frame based on a realistic instrument model. We derive the plasma bulk parameters by fitting the observations with an analytical model, using the *χ*^2^ minimization method.

Our analysis shows the following.

- The fit analysis of plasma measurements with relatively high flux (*C*_max_ > 30) estimates the plasma temperature and kappa index more accurately if it excludes measurement points with *C_i_* = 0. The corresponding analysis of measurements with low particle flux (*C*_max_ < 30) estimates the temperature and kappa index more accurately if it includes measurement points with *C_i_* = 0. Although *C_i_* = 0 is a measurement with a large uncertainty, it contains information that becomes useful when the overall signal is weak.
- Examination of the fit convergence indicates that the determination of the plasma temperature and the determination of the kappa index are interdependent. As expected, the uncertainty of the derived parameters decreases with increasing particle flux.
- The plasma density is underestimated when the particle flux is low (*C*_max_ < 100). We show that the misestimation is due to the asymmetry of the Poisson distribution and the assigned uncertainties to the data points.

Our study predicts the accuracy of the future SWA-EAS measurements. However, our methods can be applied to investigate and/or improve the accuracy of similar fitting analyses which determine kappa distribution functions. Although this paper uses the kappa distribution to describe the VDFs of plasma particles, the kappa distribution function is used to describe several other mechanisms as well (e.g., the angular scattering of particles passing through graphene and carbon foils [41,42]).

## Figures and Tables

**Table 1:** The input and the derived bulk parameters for the histograms in Figure 4.

Parameter	Input	Fit Including *C_i_* = 0 Points	Fit Excluding *C_i_* = 0 Points
*n* (cm^−3^)	7	5.4 ± 0.2	6.1 ± 0.2
*κ*	3	3.2 ± 0.2	2.3 ± 0.1
$T_{∥}$ (eV)	10	9.4 ± 0.3	12.5 ± 0.7
$T_{⊥}$ (eV)	20	19.8 ± 0.6	23.7 ± 1.0
